# Micronucleus Test of DHU001, a Polyherbal Formula, in Bone Marrow Cells of Male ICR Mice

**DOI:** 10.5487/TR.2009.25.4.225

**Published:** 2009-12-30

**Authors:** Seong-Soo Roh, Hyeung-Sik Lee, Sae-Kwang Ku

**Affiliations:** 19 Department of Herbology, College of Oriental Medicine, Korea; 29 Department of Clinical Laboratory Science, College of Health and Therapy, Korea; 39 Department of Anatomy and Histology, College of Oriental Medicine, Daegu Haany University, 290, Yugok-dong, Gyeongsan-si, Gyeongsangbuk-do, 712-715 Korea

**Keywords:** DHU001, Micronucleus test, Genotoxicity, Mice, White blood cells

## Abstract

The genotoxic effects of DHU001, a polyherbal formula were evaluated using the mouse micronucleus test. DHU001 was administered once a day for 2 continuous days by oral gavage to male ICR mice at doses of 2000, 1000 and 500 mg/kg. Cyclophosphamide was used as a known geno-toxic agent in a positive control. The appearance of a micronucleus is used as an index for genotoxic potential. In addition, the changes on the total white blood cells and differential counts on the lymphocytes, neutrophils, eosinophils, basophils and monocytes in the prepared blood smears were also conducted to observe the possible immunosuppression. The results indicats that DHU001 showed no genotoxicity effects up to 2000 mg/kg dosing levels and did not influenced on the total white blood cells and differential counts. In addition, it is also considered that there were no problems from cytotoxicity of DHU001 tested in this study because the polychromatic erythrocyte ratio was detected as > 0.41 in all tested groups.

## INTRODUCTION

DHU001 is a mixed herbal formula consisted of 7 types aqueous extracts; Ficis fructus, Liriopis tuber, Platycodi radix, Schisandrae fructus, Glycyrrhizae radix, Zingiberis rhizoma and Menthae herba and being developed for respiratory disorders. Among 7 types of herbal components of DHU001, Liriopis tuber (Park and Geon, 2003), Platycodi radix (Kim *et al*., 2004), Schisandrae fructus (Narimanian *et al*., 2005; Rhyu *et al*., 2006), Glycyrrhizae radix (Sun and Pan, 2006), Zingiberis rhizoma (Aimbire *et al*., 2007; Ghayur *et al*., 2008) and Menthae herba (Shin, 2003) has been used for treating various respiratory symptoms especially inflammatory diseases. In addition, anti-inflammatory effects on the acute inflammation of DHU001, itself were also already reported (Back *et al*., 2008).

As increase of the concern in the functional food and well being in life, the demands and consumption of functional food originated form natural sources are increased (Lee *et al*., 2003). However, the toxicological aspects about these natural origin-functional foods has been neglected because of the reasons that they has been used as various purpose for long times. Therefore, it is considered that more detailed and systemic toxicological studies should be performed to control the abuse and potential toxicities even if they have been used as traditional folk medicine.

Bone marrow cytogenetics, micronucleus test is a useful short-term technique for elucidating the mechanism as well as to identify the substances for their clastogenic and anticlastogenic activity (Renner, 1990). In Korea Food and Drug Administration (KFDA) guideline (2005-60, 2005), the genotoxicity should be tested prior to develop a new drug even though they have natural origin. Most of the mixtures having natural origin, genotoxicity has been performed using *in vivo* like micronucleus test (Kalantari *et al*., 2007).

The objective of the present study, therefore, was to obtain the genotoxic information about DHU001, a polyherbal formula being developed for respiratory disorders with the effects on the total white blood cells and their differential counts in the prepared blood to observe the possible immunosuppress, and further clarify their safety for clinical use.

## MATERIALS AND METHODS

***Animals and husbandry.*** Thirty-five male ICR mice (6-wk old upon receipt, SLC, Japan) were used after acclimatization for 10 days. The body weights of animals at receipt are ranged in 29~30 g. Animals were allocated seven per polycarbonate cage in a temperature (20~25°C) and humidity (30~35%) controlled room. Light : dark cycle was 12 h : 12 h and feed (Samyang, Korea) and water were supplied free to access. Animals were marked by picric acid.

***Test articles and formulation.*** The herbal compositions of DHU001 were listed in Table 1. Each herbal component was purchased from Cho-Heung Pharmaceutical Ind. Co. (Daegu, Korea) after confirmation of the morphology under microscopy. Approximated amounts of each herbal component was mixed (317.5 g) and boiled in 2 l of distilled water for 2 hours and than filtrated. The filtrate was decompressed using a rotary vacuum evaporator (Lab. Camp, Daejeon, Korea) and lyophilized in a programmable freeze-dryer (IlShin Lab, Daejeon, Korea). Total acquired lyophilized extracts were 34.93 g (yield 11%). Powders of extracts were stored in a desiccator to protect against light and moisture. It was well suspended up to 200 mg/ml concentration levels and appeared to be a deep brown homogenous suspension. The test article was orally administered at a dosage volume of 10 ml/kg, once a day for 2 days by oral gavage to mice; total 2000, 1000 and 500 mg/kg using distilled water as vehicle. Cyclophosphamide·$H_2O$ (CPA; Sigma, USA) was used as an identified genotoxic agents in a positive control group. CPA was dissolved in saline and once intraperitoneally administered at a volume of 10 ml/kg (70 mg/kg).

***Grouping and dosing.*** The animals were allocated into five groups 7 mice each. The fixed highest dosage level of 2000 mg/kg oral dosing was chosen in accordance to the limited highest dosage in rodent recommended by KFDA guidelines (2005), and 500 and 250 mg/kg was selected using the common ratio 2. Control negative (taken vehicle) and control positive (CPA; 70 mg/kg-single treatment) were included by recommendation of KFDA guidelines (2005) and Organization for Economic Co-Operation and Development (OECD) guidelines (1997).

***Observation of clinical signs.*** All abnormal clinical signs were recorded before and after dosing at least twice a day based on the functional observational battery test (Irwin, 1968; Dourish, 1987).

***Body weight changes.*** Body weights were measured once a day.

***Bone marrow preparation.*** All animals were sacrificed 24 h post administration using carbon dioxide, and bilateral femur was separated. Bone marrow preparations were made according to Schimid (1975). In brief, bone marrow cells were collected from aforementioned femur in 3 ml of inactivated fetal bovine serum (Gibco BRL, USA), centrifuged, and smeared on slides. Preparations were dried, and fixed by submerging in absolute methanol (for 10~20 min). Fixed slides were stained as follows;

| | |
|---|---|
| May-Grunwald stain | 3 min |
| May-Grunwald stain (1 : 1 diluted) | 2 min |
| Giemsa stain (1 : 6 diluted) | 10 min |

***Observation and recoding of micronuclei.*** Slides were randomly coded and examined under × 1000

**Table 1.** Herbal composition of DHU001 used in this study

| Herbs | Scientific name | Amounts (g) |
|---|---|---|
| Ficis fructus | *Ficus carica* Linn. | 140 |
| Liriopis tuber | *Liriope spicata* Lour. | 45 |
| Platycodi radix | *Platycodon grandiflorum* Jacq. | 60 |
| Schisandrae fructus | *Schisandra chinensis* Baill | 22.5 |
| Glycyrrhizae radix | *Glycyrrhiza uralensis* Fisch | 15 |
| Zingiberis rhizoma | *Zingiber officinale* Roscoe | 15 |
| Menthae Herba | *Mentha arvensis* Linne var piperascens | 20 |
| Total | 7 types | 317.5 |

All individual herbs were purchase from Cho-Heung Pharmaceutical Ind. Co. (Daegu, Korea) and yield 11% aqueous extracts were acquired.

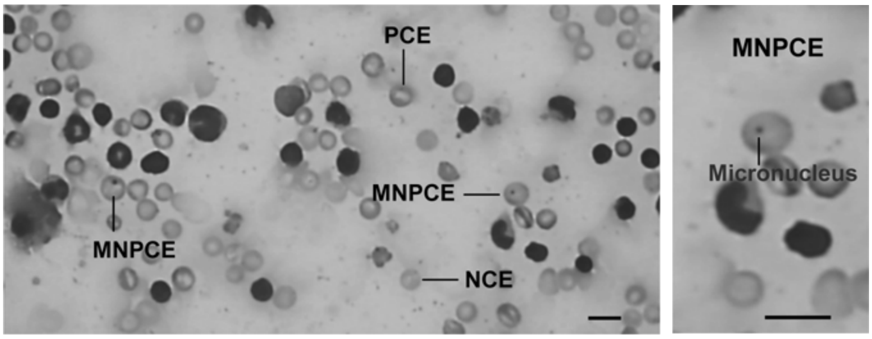

Bone marrow cell smear, CPA treated mice 05

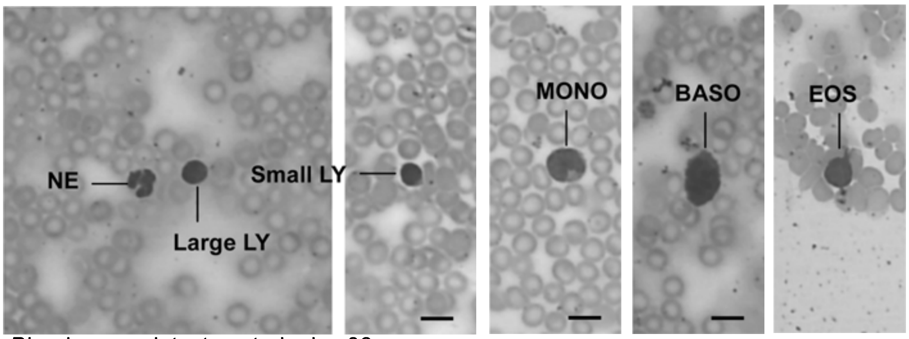

Blood smear, intact control mice 06

**Fig. 1.** Representative cytology of bone marrow cell and blood cell smears. In prepared bone marrow cell smear, polychromatic erythrocyte (PCE), normochromatic erythrocyte (NCE), PCE with one or more nuclei (MNPCE) were counted based on the above morphology. NCEs containing nucleus were not calculated. In prepared blood smear, neutrophils (NE), eosinophils (EOS), basophils (BAS), moncytes (MONO) and lymphocytes (LY) were counted based on the above morphology. Scale bars = 10 μm.

magnification by two different experts. Small round or oval shaped bodies, size of which ranging from 1/5 to 1/20 diameter of polychromatic erythrocytes (PCE), were counted as micronuclei (MN). Attention was given to discriminate micronuclei from artifacts (Fig. 1). Results were expressed as the number of MNPCEs in 2000 PCEs. Mean number of MNPCE ± standard deviation was calculated for each treatment group. In addition, PCE/(PCE + normochromatic erythrocytes (NCE)) ratio were also calculated by counting 500 erythrocytes, for detecting the possibility of cytotoxicity (Heddle *et al.*, 1984).

***Blood collection and leukocyte counts.*** Blood were collected at sacrifice from vena cava, and total blood leukocyte numbers were calculated using counting chamber, diluting pipette and Türk solution as dilution solution. In addition, cell numbers of lymphocytes, neutrophils, eosinophils, basophils and monocytes were calculated among 100 total leukocytes in smear blood samples stained with Giemsa (Fig. 1).

***Statistical analyses.*** Multiple comparison tests for different dose groups were conducted. Variance homogeneity was examined using the Levene test. If the Levene test indicated no significant deviations from variance homogeneity, the obtain data were analyzed by one way ANOVA test followed by the Sheffe test to determine which pairs of group comparison were significantly different. In case of significant deviations from variance homogeneity were observed at Levene test, a non-parametric comparison test, the Mann-Whitney U test was conducted to determine the specific pairs of group comparison. The result of statistical evaluation was regarded significantly when the P value was less than 0.05. In addition, the study was accepted when all of the PCE/(PCE+NCE) ratio are greater than 0.20 (Heddle *et al.*, 1984). Statistical analyses were carried out using SPSS for Windows (Release 14.0K, SPSS Inc., USA).

## RESULTS

***Mortalities.*** No test article-treatment related unscheduled mortalities were detected in all tested doses during the observation periods.

**Table 2.** Changes on the body weights

| Groups | Day after dosing | | |
|---|---|---|---|
| | Day 0[1)] | Day 1 | At a termination |
| Intact control | 36.44 ± 1.56 | 41.21 ± 1.53 | 36.56 ± 1.69 |
| CPA control | 36.61 ± 1.41 | 40.80 ± 1.04 | 35.97 ± 1.52 |
| DHU001 2000 mg/kg | 36.87 ± 1.99 | 40.86 ± 1.93 | 37.04 ± 1.49 |
| DHU001 1000 mg/kg | 36.53 ± 2.29 | 41.11 ± 3.33 | 36.71 ± 2.67 |
| DHU001 500 mg/kg | 37.39 ± 3.22 | 41.47 ± 3.41 | 37.41 ± 3.39 |

[a]Values are expressed as mean ± SD, g of seven mice; [1)]Start day of test article administration; All animals were overnight fasted at Day 0 and a termination, respectively.

***Clinical signs.*** During the observation period, no abnormal clinical signs were observed from DHU001 treatment.

***Body weight changes.*** No meaningful changes on body weights were detected in CPA and all tested doses of DHU001 treated groups as compared to that of control negative group (taken vehicle only) (Table 2).

***Changes on MNPCE numbers and PCE ratio.*** Significantly ($p < 0.01$) increase of number of MNPCEs among 2000 PCEs was detected in CPA 70 mg/kg a positive control group. However, no significant changes on MNPCE numbers were detected in all three different DHU001 treated groups tested as compared with vehicle control (Table 4). The PCE/(PCE + NCE) ratio in total 500 erythrocytes was detected above 0.41 in all tested groups including negative and positive control (Table 3).

**Table 3.** Changes on MNPCE numbers and PCE/(PCE + NCE) ratio observed in mice

| Group | MNPCEs /2000 PCEs | PCE/(PCE + NCE) ratio PCE + NCE = 500 cells |
|---|---|---|
| Intact control | 1.14 ± 1.07 | 0.52 ± 0.03 |
| CPA control | 59.86 ± 9.08* | 0.41 ± 0.01 |
| DHU001 2000 mg/kg | 1.00 ± 1.00 | 0.51 ± 0.01 |
| DHU001 1000 mg/kg | 1.00 ± 0.82 | 0.52 ± 0.01 |
| DHU001 500 mg/kg | 0.71 ± 0.76 | 0.51 ± 0.03 |

Values are expressed as mean ± SD of seven mice; * $p < 0.01$ compared with intact control by Mann-Whitney U test.

***Changes on the blood leukocytes.*** Except for significant ($p < 0.05$) decreases of blood total leukocyte numbers detected in CPA-injected group, no meaningful changes on the total blood leukocyte numbers and their differential counts were observed in all tested groups as compared with intact control, respectively (Table 4).

## DISCUSSION

Micronucleus assays were first introduced in the early 1970's for the examination of genotoxic activity of chemical agents (Matter and Schmid, 1971; Heddle, 1973). The procedure is based on the observation that mitotic cells with chromatid breaks or incomplete exchanges or with malfunction of the spindle apparatus suffer from disturbances in anaphase distribution of their chromatin. After telophase, a sizable portion of this displaced chromatin is not included in the nuclei of the daughter cells but forms single or multiple micronuclei in the cell cytoplasm. The frequency of the appearance of micronuclei depends both upon the rate of chromosome breakage or loss and the rate of cell division (Von Ledebur and Schmid, 1973; Heddle *et al.*, 1984). Although micronuclei can occur in almost all dividing cells, mouse bone marrow is usually the tissue used for the micronucleus test, and any agent which induces chromosomal aberrations can also produce micronuclei

**Table 4.** Changes on the blood leukocytes: total leukocyte numbers and differential counts of lymphocytes, neutrophils, eosinophils, basophils and monocytes observed in blood smear prepared

| Group[1)] | Total leukocyte numbers ($\times 10^3$cells/mm$^3$) | Proportions among 100 leukocytes (%) | | | | |
|---|---|---|---|---|---|---|
| | | Lymphocytes | Neutrophils | Eosinophils | Basophils | Monocytes |
| Intact control | 3.82 ± 1.38 | 84.57 ± 4.72 | 13.57 ± 3.36 | 0.29 ± 0.49 | 0.14 ± 0.38 | 1.43 ± 1.27 |
| CPA control | 1.80 ± 0.41* | 89.14 ± 2.85 | 9.00 ± 2.58 | 0.57 ± 0.53 | 0.00 ± 0.00 | 1.29 ± 0.49 |
| DHU001 2000 mg/kg | 3.24 ± 1.20 | 86.86 ± 5.11 | 12.29 ± 4.92 | 0.14 ± 0.38 | 0.00 ± 0.00 | 0.71 ± 0.76 |
| DHU001 1000 mg/kg | 4.22 ± 2.19 | 84.57 ± 5.97 | 14.57 ± 5.94 | 0.14 ± 0.38 | 0.00 ± 0.00 | 0.71 ± 0.76 |
| DHU001 500 mg/kg | 3.76 ± 1.16 | 85.71 ± 6.90 | 13.29 ± 7.06 | 0.14 ± 0.38 | 0.00 ± 0.00 | 0.86 ± 1.07 |

Values are expressed as mean ± SD of seven mice; * $p < 0.05$ compared with intact control by Sheffe test.

(Heddle *et al.*, 1983, 1984).

Because of its simplicity and efficacy, the micronucleus test has become a popular and useful in vivo procedure for the detection of chemically-induced chromosome damage. The number of reports from micronucleus testing has increased dramatically in the scientific literature during the past decade (Ashby, 1985), and the value of this test for examining the mutagenicity and carcinogenicity of chemicals has been emphasized, particularly when it is used in combination with other cytogenetic assays (Heddle *et al.*, 1984).

In the present study, the genotoxic effects of DHU001 were evaluated using the mouse micronucleus test with effects on the blood total leukocyte numbers and their differential counts. As the results obtained in the present study, DHU001 shows no genotoxicity effect up to 2000 mg/kg dosing levels. The highest dosage used in the present study was selected as 2000 mg/kg, recommended as the limited highest dosage in rodent by KFDA guidelines (2005), and vehicle and positive control were added according to the recommendation of KFDA (2005) and OECD (1997) guidelines. DHU001 did not influence on the blood total leukocyte numbers and their differential counts.

The PCE/(PCE+NCE) was used as index of cytotoxicity and the study was accepted when all of the PCE/(PCE + NCE) ratio are greater than 0.20 (Heddle *et al.*, 1984). The PCE/(PCE + NCE) ratio was detected as > 0.41 in all tested groups including negative and positive control in the present study. That is no problem from cytotoxicity of the tested articles used in this work.

CPA is a widely used anti-neoplasic drug, employed either alone or in combination with other products (Grochow, 1996). The parent drug is biologically inactive, however after biotransformation by microsomal enzymes a number of active metabolites capable of alkylating nucleic acids (Miyauchi *et al.*, 1990), damage the chromosomes (through generation of free-radicals) and/or alkylating the DNA thereby producing mutagenicity (El-Bayoumy, 2001) were produced. In the present study, CPA used as a positive control, and it showed a significant increases of MNPCE ratios with marked decreases of total leukocyte numbers. This indicates that the experiment protocol and the results of the present study are acceptable, and no meaningful increases of MNPCE were reported up to 2000 mg/kg of DHU001.

Based on the results, it is concluded that DHU001 shows no genotoxicity and immunosuppress effects up to 2000 mg/kg dosing levels. In addition, it is also considered that there were no problems from cytotoxicity of Polycan because the polychromatic erythrocyte ratio was estimated as > 0.41 in all tested groups.

## References

[CR1] Aimbire F, Penna S, Rodrigues M, Rodrigues KC, Lopes-Martins RA, Sertié JA (2007). Effect of hydroalcoholic extract of Zingiber officinalis rhizomes on LPS-induced rat airway hyperreactivity and lung inflammation. Prostaglandins Leukot Essent Fatty Acids.

[CR2] Ashby J (1985). Is there a continuing role for the intraperito-neal injection route of exposure in short-term rodent genotoxicity assays?. Mutat. Res..

[CR3] Back YD, Lee HS, Ku SK (2008). Effects of DHU001, a mixed herbal formula on acute inflammation in mice. Toxicol. Res..

[CR4] Dourish, C.T (1987). *Effects of drugs on spontaneous motor activity in experimental psychopharmacology* (Greenshaw, A.J and Dourish, C.T, Eds.), Humana Press, Clifton, pp. 325–334.

[CR5] El-Bayoumy K (2001). The protective role of selenium on genetic damage and on cancer. Mutat. Res..

[CR6] Ghayur MN, Gilani AH, Janssen LJ (2008). Ginger attenuates acetylcholine-induced contraction and Ca2+ signalling in murine airway smooth muscle cells. Can. J. Physiol. Pharmacol..

[CR7] Grochow, L.B (1996). *Covalent-DNA binding drugs in The chemotherapy source book* (Perry, M.C., Ed.), Williams & Wilkins, Baltimore, pp. 293–316.

[CR8] Heddle JA (1973). A rapid in vivo test for chromosome damage. Mutat. Res..

[CR9] Heddle J, Hite M, Kirkhart B, Mavournin K, MacGregor JT, Newell GW, Salamone MF (1983). The induction of micronuclei as a measure of genotoxicity: a report of the U.S. environmental protection agency gene-tox program. Mutat. Res..

[CR10] Heddle, J.A, Stuart, E. and Salamone, M.F (1984). *The bone marrow micronucleus test in Handbook of mutagenicity test procedures* (Kilbey, B., Legator, M., Nichols, W. and Ramel, C., Eds.), Elsevier, Amsterdam, pp. 441–457.

[CR11] Kalantari H, Larki A, Latifi SM (2007). The genotoxic-ity study of garlic and pasipy herbal drops by peripheral blood micronucleus test. Acta Physiol. Hung..

[CR12] Kim SS, Lee SC, Shin HD, Shin MK, Kim JH, Song HJ (2004). Studies on allergy asthma effect of Radix Platicodi. Kor. J. Herbology.

[CR13] Korea FoodDrug Administration. (2005). Testing Guidelines for Safety Evaluation of Drugs.

[CR14] Lee JE, Kim HJ, Choi EK, Chai HY, Yun YW, Kim DJ, Nam SY, Lee BJ, Ahn BW, Kang HG, Kim YB (2003). Four-week repeated-dose toxicity study on Pinellia Extract. Korean J. Lab. Anim. Sci..

[CR15] Matter B, Schmid W (1971). Trenimon-induced chromosomal damage in bone-marrow cells of six mammalian species, evaluated by the micronucleus test. Mutat. Res..

[CR16] Miyauchi A, Hiramine C, Tanaka S, Hojo K (1990). Differential effects of a single dose of cyclophosphamide on T cell subsets of the thymus and spleen in mice: flow cytofluorometry analysis. Tohoku J. Exp. Med..

[CR17] Narimanian M, Badalyan M, Panosyan V, Gabrielyan E, Panossian A, Wikman G, Wagner H (2005). Impact of Chisan (ADAPT-232) on the quality-of-life and its efficacy as an adjuvant in the treatment of acute nonspecific pneumonia. Phytomedicine.

[CR18] Organization for Economic Co-OperationDevelopment. (1997). Guideline for the Testing of Chemicals TG No. 474. Mammalian Erythrocyte Micronucleus Test.

[CR19] Irwin S (1968). Comprehensive observational assessment: Ia. A systemic, quantitative procedure for assessing the behavioral and physiological state of the mouse. Psychopharmacology.

[CR20] Park JH, Geon DG (2003). Pharmacognostical studies on the Chinese crude drug “Maig Moon Dong”. Kor. J. Pharmacogn..

[CR21] Renner HW (1990). In vivo effects of single or combined dietary antimutagens on mutagen-induced chromosomal aberrations. Mutat. Res..

[CR22] Rhyu MR, Kim EY, Yoon BK, Lee YJ, Chen SN (2006). Aqueous extract of Schizandra chinensis fruit causes endothelium-dependent and -independent relaxation of isolated rat thoracic aorta. Phytomedicine.

[CR23] Schimid W (1975). The Micronucleus Test. Mutat. Res..

[CR24] Shin TY (2003). Inhibition of immunologic and nonimmuno-logic stimulation-mediated anaphylactic reactions by the aqueous extract of Mentha arvensis. Immunopharmacol. Immunotoxicol..

[CR25] Sun HX, Pan HJ (2006). Immunological adjuvant effect of Glycyrrhiza uralensis saponins on the immune responses to ovalbumin in mice. Vaccine.

[CR26] Von Ledebur M, Schmid W (1973). The micronucleus test - methodological aspects. Mutat. Res..

